# An investigation into the optimal number of distractors in single-best answer exams

**DOI:** 10.1007/s10459-015-9652-7

**Published:** 2015-11-23

**Authors:** James M. Kilgour, Saadia Tayyaba

**Affiliations:** Institute of Medical Education, School of Medicine, Cardiff University, The Cochrane Building, Heath Park Campus, Cardiff, CF14 4XN UK

**Keywords:** Assessment, Testing, Written examination, Examination reliability, Examination quality, Single-best answer exams, Undergraduate medical education

## Abstract

In UK medical schools, five-option single-best answer (SBA) questions are the most widely accepted format of summative knowledge assessment. However, writing SBA questions with four effective incorrect options is difficult and time consuming, and consequently, many SBAs contain a high frequency of implausible distractors. Previous research has suggested that fewer than five-options could hence be used for assessment, without deterioration in quality. Despite an existing body of empirical research in this area however, evidence from undergraduate medical education is sparse. The study investigated the frequency of non-functioning distractors in a sample of 480 summative SBA questions at Cardiff University. Distractor functionality was analysed, and then various question models were tested to investigate the impact of reducing the number of distractors per question on examination difficulty, reliability, discrimination and pass rates. A survey questionnaire was additionally administered to 108 students (33 % response rate) to gain insight into their perceptions of these models. The simulation of various exam models revealed that, for four and three-option SBA models, pass rates, reliability, and mean item discrimination remained relatively constant. The average percentage mark however consistently increased by 1–3 % with the four and three-option models, respectively. The questionnaire survey revealed that the student body had mixed views towards the proposed format change. This study is one of the first to comprehensively investigate distractor performance in SBA examinations in undergraduate medical education. It provides evidence to suggest that using three-option SBA questions would maximise efficiency whilst maintaining, or possibly improving, psychometric quality, through allowing a greater number of questions per exam paper.

## Background

Multiple-choice questions (MCQs) have been used as a form of knowledge assessment since the early twentieth century (Trewin 2007). They are now commonplace in both undergraduate and postgraduate medical examinations (Shumway and Harden 2003), as they are standardised, equitable, objective, cost effective, reliable and discriminatory (Al-Rukban 2006). In addition, they can be used to assess both factual recall and more complex cognitive functions such as diagnostic skill, evaluation and reasoning (Epstein 2007). They also enable the assessment of a broad range of content, as each exam paper can contain a large number of items. This makes the MCQ format particularly suited to summative final examinations, and also national licensing tests (Epstein 2007; van der Vleuten 2000). The major drawback to the MCQ format however, is that high quality questions are difficult, time-consuming and costly to write (Al-Rukban 2006; Epstein 2007; Shumway and Harden 2003).

The incorrect options to a MCQ are known as distractors; they serve the purpose of diverting non-competent candidates away from the correct answer (Burton et al. 1991). Lowe (1991) noted that “the mark of a good question is often the quality of the distractors” (Lowe 1991, p. 780), and this reflects the importance of these incorrect options for discrimination between examinees. As the number of high quality, functional distractors increases, so does item difficulty and discrimination (Haladyna and Downing 1988, 1993; Rogausch et al. 2010; Tarrant et al. 2009). Undergraduate medical examinations typically use a specific form of MCQ, known as single-best answer (SBA), where all of the incorrect distractors still contain some element of truth, but to a competent candidate are perceptibly inferior to the one correct response. The process of generating distractors for SBA questions is challenging and labour intensive, and this often results in unconvincing distractors being chosen (Al-Rukban 2006). It has been further argued by the literature that in most cases, there is a natural limit to the number of plausible distractors possible for any given topic, and that this limit is generally less than the four conventionally used in medical school SBA papers (Haladyna and Downing 1993; Haladyna et al. 2002).

There is substantial evidence to suggest that across diverse academic disciplines, the majority of MCQs used in examinations contain a high proportion of non-functioning distractors. These are distractors which either have been selected by <5 % of the target cohort (Cizek and O’Day 1994; Delgado and Prieto 1998; Haladyna and Downing 1993; Rogers and Harley 1999; Shizuka et al. 2006; Sidick et al. 1994; Tarrant et al. 2009), or show negative discrimination statistics (Measured Progress 2014). A key psychometric investigation by Haladyna and Downing (1993) found that in a two hundred item MCQ exam administered to US physicians, no items had four functioning distractors, and only 8.4 % of questions had three (Haladyna and Downing 1993). Given that non-functioning distractors do not significantly add to the difficulty, discrimination or reliability of exam papers (Haladyna and Downing 1988; Rodriguez 2005), it was therefore hypothesised that the removal of poor quality distractors from SBA questions, to instead use a reduced-option format, should not have an impact on exam quality. This hypothesis has been supported by numerous other studies; the earliest involved mathematical modelling (Bruno and Dirkzwager 1995; Grier 1975; Lord 1977; Tversky 1964), whilst later studies went on to use experimental approaches. These investigations revealed that a reduced-option MCQ format is equivalent or superior to the conventional format in terms of the number of functioning distractors (Trevisan et al. 1991; Wakefield 1958), exam reliability (Baghaei and Amrahi 2011; Costin 1972; Delgado and Prieto 1998; Rogers and Harley 1999; Ruch and Charles 1928; Shizuka et al. 2006; Sidick et al. 1994; Trevisan et al. 1991, 1994), item discrimination (Baghaei and Amrahi 2011; Costin 1972; Crehan et al. 1993; Delgado and Prieto 1998; Owen and Froman 1987; Shizuka et al. 2006; Trevisan et al. 1991) and difficulty (Baghaei and Amrahi 2011; Crehan et al. 1993; Delgado and Prieto 1998; Landrum et al. 1993; Owen and Froman 1987; Shizuka et al. 2006; Sidick et al. 1994). Several systematic reviews have also provided evidence to confirm these findings (Aamodt and McShane 1992; Haladyna and Downing 1989; Haladyna et al. 2002; Rodriguez 2005; Vyas and Supe 2008). Furthermore, student perceptions of a reduced-option MCQ model have also been investigated with positive results (Owen and Froman 1987).

This literature shows that there is evidence to support a reduced-option MCQ format, however, to date, little empirical research has been produced to investigate this phenomenon in medical education. Whilst some early studies involved the assessment of postgraduate medical training (Cizek and O’Day 1994; Cizek et al. 1998; Haladyna and Downing 1988, 1993), and more recent studies have involved nursing (Tarrant et al. 2009) and dental students (Kolstad et al. 1985), there have only been three studies involving undergraduate medical students. Two of these studies were of limited scope and are therefore of limited generalisability (Schneid et al. 2014; Swanson et al. 2008). Rogausch et al. (2010) conducted the most comprehensive study, which involved a sample of 737 questions from the Swiss Federal graduation exam. At the 5 % frequency of selection level, only 2.8 % of questions in this sample had four functional distractors. The authors also modelled the effect of reducing the number of options from five to three, finding that this change resulted in only a slight increase in mean percentage correct, whilst discrimination was almost unaffected. However, reliability was markedly decreased, which is in contrast to the previous literature. It is important to note, nevertheless, that the assumption was made that candidates who chose the least functional distractors would otherwise have chosen the correct answer, and therefore they reallocated these candidates as such (Rogausch et al. 2010).

Despite evidence from the wider educational measurement literature, there is still a lack of high-quality, generalizable and comprehensive research specific to undergraduate medical education to support a reduction in the number of distractors per SBA item. Furthermore, the evidence which has been produced in this area in inconsistent with the previous literature (Rogausch et al. 2010). There is therefore a clear need for elucidation and clarification in this field. This paucity of evidence may explain the reluctance of medical schools to make changes to the current SBA model (Tarrant et al. 2009). This is likely further compounded by the fears of assessment writers, who may believe that three-option questions will increase successful guessing by non-competent candidates (Schneid et al. 2014). The overall objective of this research study was therefore to investigate SBA distractor functionality from an undergraduate medical assessment perspective.

## Methodology

We used a mixed-methods approach for this study, involving both statistical analysis of exam data, as well as administering a survey containing a mixture of Likert and free-text responses. Ethical approval was granted for this research by the *Cardiff University School of Medicine Research Ethics Committee* in December 2014.

### Sample

Four past examination papers from the 2013/2014 Cardiff University undergraduate medical programme were scrutinised for this study, which provided a sample of 480 five-option SBA questions. A mean of 269 students sat each of these exam papers, which hence provided a sufficiently large evidence base for psychometric modelling (Schuwirth and van der Vleuten 2011). These papers were chosen due to their high reliability, which indicated an existing high level of quality, in addition to their wide-ranging coverage of general medicine and medical and surgical specialities, including sociology, ethics, pharmacology and epidemiology.

The survey component of this study used a sample of 327 students from Cardiff University during the 2014/2015 academic year, including 252 fourth year students, and 75 students undertaking an intercalated degree after the third or fourth year. Students for this component of the study were recruited through generic emails sent to all students in the target cohort, and through social media (i.e. Facebook). No financial or other incentives were offered to students to encourage participation.

### Measures

Several measures of exam quality were used in the modelling aspect of this study. These measures were reliability, difficulty, mean item discrimination and pass rates. Exam reliability, or internal consistency (Wells and Wollack 2003), is one of the most important psychometric properties of an exam paper, as it reflects the precision and consistency of the measurement made by an exam, and hence the reproducibility of its outcome (van der Vleuten 2000). In this study, we measured reliability using Cronbach’s Alpha, as it only requires data from one sitting of the exam paper under evaluation (Tavakol and Dennick 2011). In this study, we also measured exam difficulty, which is equivalent to the exam’s mean percentage mark, and item discrimination, using the mean of each item’s point-biserial correlation coefficient (Rpbis). In addition, we also sought to measure the student acceptability and educational impact of a reduction in the number of options per question, which is essential to consider (van der Vleuten and Schuwirth 2005).

### Procedure

The study followed a two-step procedure, where the first step was to determine the frequency of non-functioning distractors across the sample of exam papers. This was achieved by analysing the frequency of selection at the below 5 % level. This data was calculated using the ITEMAN psychometric software (Assessment Systems Corporation 2013).

The second step was to model the effects of the reduced-option models on the psychometric measures of each exam paper. The impact on each measure was assessed twice; firstly, for a reduction from five options per question to four, and secondly, for a reduction from five options to three. This analysis was firstly achieved by using the frequency data calculated by ITEMAN to eliminate the least and second least functional distractor for each question. In cases where there was a tie in selection frequency between multiple distractors, elimination was determined by comparing the Rpbis; a more-negative point-biserial was considered as indicating a higher level of functionality. The frequency of students who had selected one of the eliminated distractors was then randomly reassigned to one of the remaining options. The rationale for the random redistribution of these students is based on the assumption that they were most likely guessing the answer, having chosen the least plausible response to the question. Random redistribution can hence be considered a legitimate simulation (Tarrant et al. 2009).

Once elimination of non-functioning options had occurred, various data sets were then created using IBM SPSS Statistics (IBM Corporation 2014), to investigate the impact of the reduction in the number of distractors on the various exam attributes. The statistical significance of any changes in mean values were determined by computing relevant tests of significance. Furthermore, one-way analysis of variance (ANOVA) tests were carried out to compare the differences in mean scores between ability groups (low, average and high performance) for each year group and for each of the three exam paper models.

The questionnaires for the survey component of this study were distributed online through the SurveyMonkey™ platform (SurveyMonkey Inc. 2014) (see “Appendix” section). Participation was voluntary and anonymous. Once all survey responses were collected, analysis was then carried out for quantitative data using Microsoft Excel (Microsoft Corporation 2011), and for qualitative data using the ATLAS.ti software (Scientific Software Development GmbH 2012). This is a specialist qualitative research program, which facilitates the aggregation and comparison of data from across responses through the use of a coding hierarchy, which was developed through an inductive, thematic approach (Bradley et al. 2007).

## Results

An analysis of the performance of the examination papers included in this study is shown below (Table 1). Overall, all of the papers demonstrated reasonable attributes in terms of reliability and individual question performance.

**Table 1:** Analysis of overall examination performance for all 3 years

Statistic	Y03	Y04	Y05
Alpha reliability^a^	0.81	0.82	0.79
Average score	95.14	144.98	87.94
Maximum possible score	140	200	140
Standard deviation (SD)	11.02	12.88	11.01
Range of scores	61–126	108–172	63–125
Number of items	140	200	140
Average percentage correct	68 %	72 %	63 %
Average item discrimination (Rpbis)^b^	0.16	0.14	0.14
Number of candidates	262	278	273

^a^Alpha reliability ranges between 0 and 1 (i.e. no consistency to perfect internal consistency). The desirable range for high stake assessments is 0.8–0.89. The higher the stakes of the examination, the higher the value of the alpha is required to be in order to ensure a high degree of confidence in pass/fail decisions

^b^Rpbis ranges between −1 and 1 (i.e. negatively discriminatory to perfectly discriminatory). In high stakes examinations, it is desirable to have an Rpbis approaching 0.20, as this indicates a high level of discrimination between competent and non-competent candidates

### Analysis of question performance

In total, 480 question items and 1920 distractors were examined in this study. The analysis of question performance (Table 2) reveals that across the papers included in this study, the average number of functional distractors per question (those which were chosen with a frequency equal to or >5 % of the cohort) was 1.82.

**Table 2:** **Number of** functional distractors per item

Number of functional distractors per question	Y03	Y04	Y05	Overall
Zero	18 (12.9 %)	39 (19.5 %)	11 (7.9 %)	68 (14.2 %)
One	35 (25.0 %)	59 (29.5 %)	33 (23.6 %)	127 (26.5 %)
Two	43 (30.7 %)	61 (30.5 %)	55 (39.3 %	159 (33.1 %)
Three	32 (22.9 %)	30 (15.0 %)	30 (21.4 %)	92 (19.2 %)
Four	12 (8.6 %)	11 (5.5 %)	11 (7.9 %)	34 (7.1 %)
Average	1.89	1.58	1.98	1.82

Overall, only 34 questions (7.1 %) of the 480 included in this study contained four functional distractors, whilst 92 (19.2 %) contained three. The greatest proportion of questions, 159 (33.1 %), had two functional distractors, whilst many questions contained only one (127, 26.5 %). Finally, 68 questions (14.2 %) contained no functional distractors, and were therefore completely non-discriminatory.

### Analysis of distractor performance

Analysis of the performance of the 1920 distractors included in this study reveal that 1062 (55.3 %) of the distractors were non-functional, with 341 of the distractors (17.8 %) being so implausible that they were never chosen. Of the 858 (44.6 %) functional distractors analysed, only 206 (10.7 %) were chosen by more than 20 % of the examinee cohort (Table 3).

**Table 3:** Breakdown of individual distractor performance grouped into categories by frequency of selection (all years combined)

Distractor functionality by frequency of selection (%)	Number (%)
0	341 (17.8)
<5	721 (37.6)
5–10	374 (19.5)
11–20	278 (14.5)
>20	206 (10.7)

### Modelling the effects of the question models on exam attributes

The results of the simulation of the different exam paper models are presented in Table 4, showing the calculated effect on the selected psychometric measures. These results reveal that the mean percentage mark for each paper would have increased by 1 % following a change from the five-option paper to the four-option version, and would have increased by a total of 3 % if the three-option model had been used. In order to test the statistical significance of these changes, a series of paired samples *t* tests were carried out, using the original five-option model as a baseline. For all years, the changes in difficulty between the five-option version of each paper and the four-option version, and the four-option version and the three-option version were all statistically significant, with *t* values >10 points, narrow confidence intervals (CI) and significance in the expected direction (*p* < 0.001).

**Table 4:** Effect of reducing the number of options per item on important exam attributes (five, four and three option models)

Year	Psychometric attribute	Five-option model	Four-option model	Three-option model
Y03	Mean % correct	68 %	69 %	71 %
	Mean Rpbis	0.16	0.16	0.15
	Alpha reliability	0.81	0.81	0.82
	Number of fails	2	2	2
Y04	Mean % correct	72 %	73 %	75 %
	Mean Rpbis	0.14	0.17	0.16
	Alpha reliability	0.82	0.82	0.82
	Number of fails	1	1	1
Y05	Mean % correct	63 %	64 %	66 %
	Mean Rpbis	0.14	0.14	0.13
	Alpha reliability	0.79	0.80	0.80
	Number of fails	0	0	0
Average	Mean % correct	68 %	69 %	71 %
	Mean Rpbis	0.15	0.16	0.15
	Alpha reliability	0.81	0.81	0.81

Furthermore, ANOVA testing demonstrated that the difference in mean total score between different ability groups, for each paper, remained statistically significant (*p* < 0.001) (with substantially large f values and 95 % CI), regardless of which exam model was used.

Mean item discrimination (Rpbis) changed in a less linear fashion than exam difficulty. For the year three paper, discrimination was equivalent across the five-option and four-option exam models at 0.16, but it decreased by a statistically significant degree to 0.15 for the three-option model (*p* < 0.017 five-option to three, *p* < 0.004 four-option to three; upper 95 % CI of the difference for both −0.01). For the two papers in year 4, discrimination increased when the four-option model was employed, with the point-biserial correlation significantly increasing from 0.14 to 0.17 (*p* < 0.001; upper 95 % CI 0.03). Discrimination then significantly decreased when the three-option model was employed, to 0.16 (*p* < 0.001; upper 95 % CI −0.01), although discrimination for the three-option model remained significantly higher than for the original five-option version (*p* < 0.001; upper 95 % CI 0.01). Finally, for the year 5 paper, discrimination remained constant between the five-option and four-option models (0.14), but decreased (to 0.13) for the three-option model. No statistical testing was performed for this change however, as the standard error of the difference was zero.

The mean changes in exam reliability and fail rates were also modelled for the three exam formats. Overall, reliability was not significantly affected by the change in the number of options per item, although some slight increases (as the number of options decreased) were observed (year 3 and year 5). The fail rate of each exam also remained constant regardless of the number of options, as determined by the panel-agreed standard set mark for the five-option paper.

### Survey responses

#### Quantitative data

There were 108 responses to the survey questionnaire, equalling a response rate of 33.0 %. The first quantitative question (Q3; 103 responses), asked students to assess how fair they believed SBA exams in medical school to be, using a five-point Likert scale (with options ranging from ‘not at all’ to ‘completely’). A high proportion of respondents (83; 80.6 %) indicated high agreement (‘completely’ or ‘to a fair extent’) with the statement that SBA exams are a fair method of knowledge assessment, whilst 18.4 % (19) of respondents indicated low agreement (‘average’ or ‘to some extent’), and just 1 % (1) of students indicated disagreement (‘not at all’).

**Fig. 1:**
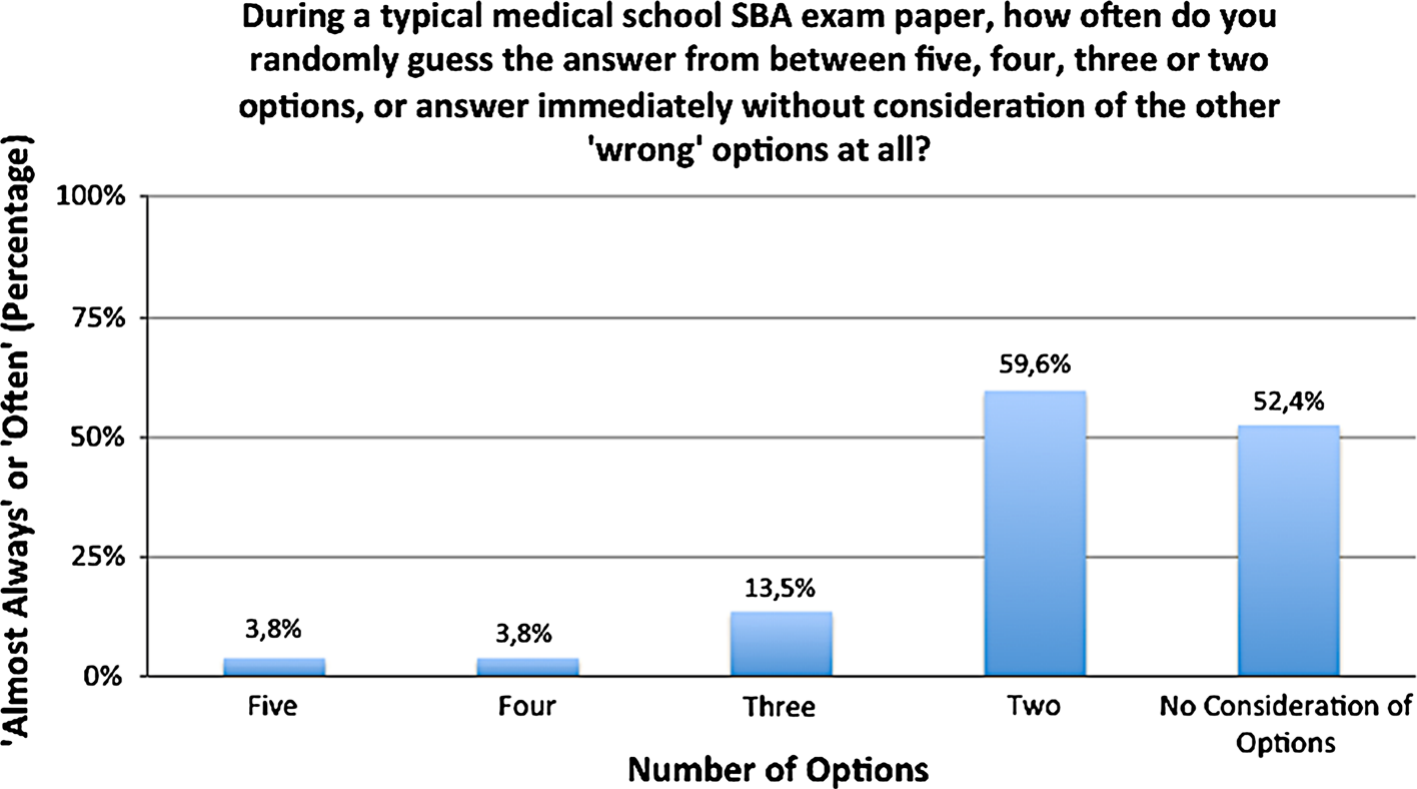
Student self-perceived ability to eliminate distractors (N = 104)

The next question (Q4; Fig. 1) was split into five sub-questions, with the purpose of examining student perceptions of the functionality of SBA distractors, in terms of their self-perceived ability to eliminate them (104 responses). The results show a positive trend towards students believing themselves able to successfully eliminate down to a single distractor, or to answer the question without consideration of the distractors at all, a high proportion of the time. Only 3.8 % (4) of respondents stated that they often randomly guess the correct answer from the five options available (‘often’ or ‘almost always’), whilst 59.6 % (62) of respondents stated by the same measure that they often randomly guess the correct answer from just two remaining options. Likewise, 52.4 % (54) of respondents answered that they are often able to answer the question immediately, without consideration of the other options at all.

#### Qualitative data

Question five of the survey explored the strategies used by students to respond to summative SBA examinations (88 responses). The responses reveal that the majority of students felt that they often use rote memorisation when preparing for these type of assessments, with 52 (59.1 %) responses indicating rote memorisation. The use of practice questions and past papers was another often highlighted surface-learning revision strategy, with 37 (42 %) responses indicating their use, whilst other unspecified surface-learning approaches were referred to in 16 (18.2 %) responses. In contrast to this, some survey respondents also mentioned that they used deep-learning strategies for SBA exam revision. This included 26 (29.5 %) responses referring to unspecified deep-learning approaches, whilst specified approaches included writing notes (12; 13.6 %), group discussion (2; 2.3 %), and use of explanatory videos (2; 2.3 %). Many respondents indicated the use of both surface-learning and deep-learning approaches (44; 50 %).

Question six of the questionnaire investigated the strategies employed by medical students to respond to five-option SBA assessments (86 responses). Responses were broadly categorised into five response strategies, with the most often cited strategy being the use of the ‘process of elimination’ (63; 73.3 %).

The final question (Q7) of the survey related to the students’ appraisal of a possible change from five-options to four or three (87 responses). Of these responses, 43 (49.4 %) contained elements of positivity towards the proposed change, including twelve referring to increased efficiency (13.8 %), nine relating to the change being a more effective use of time (10.3 %) and six relating to the positive effect on cost effectiveness (5.7 %). One respondent summarised these ideas in their response:

As most of the time I am able to narrow it down between two answers and usually one or two of the five options are quite obviously wrong then reducing it to 3 or 4 would seem reasonable (Respondent 75)

Other positive responses also cited a positive effect on learning (2), an increase in assessment quality (2), an increase in fairness (2), a reduction in stress (1), suitability for dyslexics (1) and finally that a reduced option model would be a better reflection of real-life clinical practice (1).

Just over three-quarters of the responses received from students contained elements of negativity towards the proposed change in SBA format (68; 78.2 %). Many of these responses indicated a belief that the five-option format was the best (11; 12.6 %), whilst some students concluded that whilst they believed that a four-option model would be adequate, three-options per question would be too few (10; 11.5 %). Many students also felt that using a reduced option model would lead to unfairness, as the resulting exam would be ‘easier’ (13; 15 %) whilst a high number cited the theoretical increase in probability of successful guessing as a source of unfairness and reduced examination rigour (19; 21.8 %). Other negative responses expressed by students included that reducing the number of options would increase the exam’s difficulty (3), would have a negative effect on student learning (2), would require adjustment (i.e. ‘getting used to’) (2), may be more stressful (2), and may prepare students less for clinical practice (4).

## Discussion

This study set out to investigate the optimal number of distractors in SBA assessments. The results of this study reveal that non-functioning distractors may be commonplace in high-stakes, summative, medical school assessments. Across the sample of four papers that we analysed, only 7.1 % of questions contained four functional distractors, whilst 73.8 % of questions contained two or fewer. These results are in line with what was expected, supporting the theory of Haladyna and Downing that for most topics, there is a natural limit to the possible number of plausible distractors (Haladyna and Downing 1993; Haladyna et al. 2002). These findings also match those reported by other studies in the healthcare education literature (Rogausch et al. 2010; Tarrant et al. 2009). As non-functioning distractors do not add to the discrimination power of the question, it is hence likely that in most circumstances, three-option questions should be sufficient (Rodriguez 2005; Tarrant et al. 2009). Overall, as has been previously suggested, it is the quality, not the quantity, of distractors which determines assessment quality (Haladyna and Downing 1989). It is concerning however that 14.2 % of the questions in our study were found to not contain any functional distractors, and were therefore not discriminatory. A similar finding was also reported in a study of nursing assessments (Tarrant et al. 2009), suggesting that this may be a widespread problem. It is therefore important that question writers continually refine and improve poorly functioning questions.

Our simulation of the various exam models has furthermore demonstrated evidence to support that a three-option format may be optimal. Consistent with previous healthcare research, exam reliability across all four papers investigated did not change (Rogausch et al. 2010; Tarrant et al. 2009), whilst any changes in mean item discrimination were statistically significant yet negligible (Rogausch et al. 2010; Swanson et al. 2008). Contrary to some previous research however, reporting a fail-to-pass reclassification rate of 1.9 % (Tarrant et al. 2009), in our study, pass rates also remained constant. It should be noted however that the aforementioned study involved low stakes classroom tests, and this may explain the difference. The only psychometric property that we found changed to an important degree between item models was the average percentage mark, which increased consistently by 1 % when a four-option model was employed, and by 3 % when a three-option model was used. These findings are again consistent with the previous healthcare literature (Rogausch et al. 2010; Swanson et al. 2008; Tarrant et al. 2009), reflecting a trend that fewer options per question is associated with a small yet significant increase in percentage correct, resulting in marginally easier exams. This decrease in difficulty could easily be accounted for however during the standard setting process, by adopting an increased pass threshold (Rogausch et al. 2010).

To further probe the impact of various distractor models on performance across ability groups, we carried out a series of comparative ANOVA tests across each assessment. Our findings indicated that despite the reduction in difficulty, the differences in average total score between performance groups remained statistically significant, indicating that performance remained discriminative.

The findings of this new study offer clear support to the notion that a three-option SBA model may be optimal for summative knowledge assessment in undergraduate medical education, maximising efficiency whilst maintaining examination quality and rigour. Fundamentally, the three-option model would allow for an increase in the number of questions per unit time, as has been reported by other studies (Aamodt and McShane 1992; Owen and Froman 1987; Rodriguez 2005; Schneid et al. 2014; Swanson et al. 2008; Vyas and Supe 2008). One such study involving medical and pharmacy students in fact suggested a saving of 8 s per question for three-option MCQs over their five-option counterparts (Schneid et al. 2014). This is significant, as increasing the number of questions per exam paper would facilitate broader content coverage, enhancing reliability, and potentially validity (Schneid et al. 2014; Wells and Wollack 2003). This increase may compensate for any initial reduction in reliability caused by the use of a reduced-option format (Schneid et al. 2014; Wells and Wollack 2003), and may in fact actually lead to an improvement in examination quality over the status quo. Furthermore, the burden to question writers would be reduced, allowing them to focus their time on selecting a smaller number of more plausible distractors, consequently increasing quality whilst maximising cost and time efficiency (Delgado and Prieto 1998).

Analysis of the data collected from the survey questionnaire additionally offers further evidence to support our conclusions. Notably, 59.6 % of respondents perceived themselves as often needing to guess the correct answer at random from two remaining options, whilst by the same measure just 3.8 % felt that they often had to guess the answer from between four or five options. This data trend reveals that student experience closely corresponds with our statistical data; it is clear that the majority of the time, students are able to use the process of elimination to rule out at least two options. We also collected data on the strategies that students use to learn and revise for SBA assessments, which indicated a preference for rote memorisation (59.1 %). If a three-option model were to be adopted, it is unlikely that student-learning styles would significantly change, most likely remaining highly dominated by surface-learning approaches.

Student appraisal of the change in SBA format was mixed; 49.4 % of responses contained elements of positivity towards the proposed new format, whilst 78.2 % of responses contained negativity. A significant proportion of students (21.8 %) expressed fear that a reduced number of options per question would increase the probability of successful guessing, hence leading to unmerited passes. This attitude towards the three-option format is surprising, given that the previous literature had suggested strong student support (Owen and Froman 1987), although similar fears have been discussed in association with educators and question writers (Schneid et al. 2014). If the three-option format were to be implemented, it would be important to adequately address these fears, in order to mitigate stakeholder resistance.

### Strengths and limitations

The primary limitation of this study is that it only analysed the performance of a sample of 480 SBA questions from a single UK medical school. Despite the high concordance of this study’s results with the findings of other key papers in the literature, generalisability to all medical assessments cannot be assumed. However, as at least a proportion of the exam questions in this sample came from the UK-wide *Medical Schools Council Assessment Alliance* (Medical Schools Council 2015), and the university’s assessments are subject to regulatory validation, the case for generalisability, at least within the UK, can be argued. It would still be valuable however for future research to be carried out to confirm these findings in other schools internationally, before a change in examination format could be reliably implemented. This is particularly pertinent given that our study was based on theoretical simulation, as opposed to experimental investigation.

The findings of the questionnaire survey are also subject to some limitations, including the low response rate of 33 % and the recruitment of students from a single year group, at a single medical school. Together, these factors mean that it is hard to gauge the generalisability of the survey’s findings to other populations.

Despite these limitations, this is the first comprehensive and generalizable study to investigate distractor performance in SBA examinations in the context of undergraduate medical education. Our findings have important implications for test development, including question writing training, time and resource use, and for examination quality, with our findings potentially leading to an economical improvement over the status quo. The growth in the use of SBAs as part of progress testing and computerised marking, and the corresponding need for medical schools to invest in the infrastructure to support this, indicate that further research is urgently needed. This research should aim to confirm our findings in other assessment data sets, and use more rigorous item response theory models. Finally, it would be invaluable to qualitatively explore the perceptions of test developers, question writers and assessment groups towards a reduced-option SBA model as part of a pragmatic approach to enhancing medical school assessment programmes.
